# Refractive index sensor based on fano-magnetic toroidal quadrupole resonance enabled by bound state in the continuum in all-dielectric metasurface

**DOI:** 10.1038/s41598-024-54579-8

**Published:** 2024-02-19

**Authors:** Javad Maleki, Davood Fathi

**Affiliations:** Department of Electrical and Computer Engineering, Tarbiat Modares University (TMU), Tehran, Iran

**Keywords:** Optical sensors, Terahertz optics

## Abstract

For the first time, an all-dielectric metasurface ultra-sensitive refractive index (RI) sensor with very high quality factor (QF) and figure of merit (FOM), with Fano-magnetic toroidal quadrupole (MTQ) resonance enabled by bound state in continuum (BIC) in terahertz (THz) region was designed. Furthermore, the MTQ resonance in the THz due to a distortion of symmetry-protected bound states in the continuum in the designed structure was investigated. Also, to achieve the dark mode, a combination of three methods including (i) breaking the symmetry, (ii) design of complex structures, and (iii) changing the incident angle was utilized. The broken symmetry in the structure caused a new mode to be excited, which is suitable for sensing applications. The designed metasurface was able to sense a wide range of RI in MTQ resonance, where its properties were improved for the value of sensitivity (S) from 217 GHz/RIU to 625 GHz/RIU, for FOM from 197 RIU^–1^ to 2.21 × 10^6^ RIU^–1^ and for QF from 872 to 5.7 × 10^6^.

## Introduction

Terahertz frequency range has received much attention in recent years for wideband absorber, antennas, wireless communications, magnetic wire, surface plasmons, tunable double plasmon-induced transparency, graphene based devices and sensing applications such as biomedical sensors, chemical detectors, gas sensing and so on^1–13^. The primary advantage of employing this frequency range for sensor applications is the low photon energy and effective interaction with biomolecules^14^. Biomolecules possess a distinctive absorption spectrum in the terahertz region, characterized by strong absorption and dispersion. The majority of biomolecules exhibit less energy and non-ionizing properties in this frequency range compared to the X-ray spectrum, primarily due to their rotational and vibrational transitions. Consequently, terahertz waves are well-suited for measuring biomolecular samples with high sensitivity (S) and ease of operation^2,6,15^. Utilizing intrinsic dielectric materials with low losses and thermal conductivities in the terahertz region instead of metals, with strong confined electromagnetic field, provides a high quality factor (QF) which enhances the sensor parameters such as S and figure of merit (FOM)^1,2,16–20^. Due to lower non-radiative losses in dielectric materials compared to metals, the likelihood of Fano resonance (FR) increases, leading to easy excitation of Mie multipoles and an increase in resonance intensity. This results in a reduction of the Fano transmission spectrum width, which is favorable for sensor applications^21,22^. FR is essentially an asymmetric resonance that arises from the interference between continuous and discrete states, leading to transmission and reflection spectra. Breaking symmetry in the structure is a general method for achieving FR, which results in the failure of symmetry in the displacement current distribution and the creation of a dark mode^23–25^. According to the Mie theory, the resonance caused by an electromagnetic multipole can generate electric or magnetic responses in a metasurface structure, which may consist of electric dipole (ED), magnetic dipole (MD), electric toroidal dipole (ETD), magnetic toroidal dipole (MTD), electric quadrupole (EQ), magnetic quadrupole (MQ), magnetic toroidal quadrupole (MTQ), octupoles and higher order multipoles. These responses can lead to negative effective permittivity or permeability coefficients at the resonance frequency^12,22,26–28^. In 1957, Zel’ Dovich introduced the first member of the toroidal multipole’s family, and showed that the behavior of these multipoles are different from that of electric and magnetic ones^29^. The first member of this family, ETD, originates from the electrical poloidal currents that flow on a torus surface along its meridians. An electric poloidal current is generated by a set of MDs forming a closed loop, connecting the head to the tail, while an MTD mode is formed by loops of electric field rotating around a vortex magnetic field. ETDs, MTDs, and higher order multipoles can be derived from the multipole expansion in cartesian coordinates^30–32^. Toroidal multipoles were first discovered in the microwave frequency region, and they have since been reported in metal and dielectric structures in a variety of other frequency regions^33–36^. The toroidal response can be excited in the light mode due to structural engineering or in the dark mode due to symmetry breaking^35,36^.High-order toroidal modes are referred to the higher-order toroidal moments and anapole states in all-dielectric photonics^31^. These modes are characterized by their complex field distributions^31,37^. Moreover, they can exhibit unique properties and functionalities in various applications. They can also be used for mechanically tunable polarization beam splitting^38^. These modes have potential applications in numerous fields, such as high-sensitivity sensing, light manipulation, and the development of advanced photonic devices^39^.

To achieve a dark mode, the structure should not possess mirror symmetry, as it leads to symmetrical current distribution. Alternatively, to attain a dark mode, adjusting the incidence angle and employing structural engineering can be employed^40–43^. A high QF, which means low bandwidth, is desirable for sensor applications. For operating well in noisy environments, the sensor should be able to detect a tiny shift in the frequency spectrum owing to a change in the refractive index (RI) of the media^44,45^. With appropriate structural design, a coupling between dark and light modes can be created, enabling the input wave to couple with the dark mode. The reflection and transmission spectra of such a structure exhibit FR, which is significantly narrower than other resonances. Breaking symmetry in the structure is a general method for achieving FR, which results in the failure of symmetry in the displacement current distribution and the creation of a dark mode^46,47^. The dark mode's lifetime is adjustable by changing the asymmetry parameter and creating a stronger FR inside the structure^48,49^. The basic principle of FR is interference and weak connection between light and dark modes. In addition to structural symmetry breaking and changing the incidence angle, another way for reaching light and dark modes' hybridization and consequently occurring the FR, is to design complex and hybrid structures^50–53^. Due to the unique properties and capabilities of nano disk resonator metasurface-based sensors, they have emerged as a promising platform for sensing applications. These sensors are considered as a highly sensitive platform for biosensing and surface-enhanced sensing applications^54^. A bound state in the continuum (BIC) is a resonant mode confined within a material that does not couple with the radiating channels outside the system. These states have infinite lifetimes, or Q-factors, despite having frequencies that are degenerate with the surrounding continuum of radiative/scattering channels^55^. A novel approach to confine light with infinite lifetimes, without any radiation is using BICs in all-dielectric metasurfaces^56^. The coexisted localized states, BICs, with extended waves inside the continuous spectrum range, possessing infinite lifetimes without any radiation^57^. These states have been observed in various wave contexts, including electromagnetic waves^55^, acoustic waves and elastic waves in solids^58^. They have been also studied in diverse material systems such as photonic crystals^59^, optical waveguides^60^, quantum dots^61^, and topological insulators^62^. Recent developments in metasurface have led to the creation of multiple papers such as ultrasensitive dual-band terahertz metasurface sensors based on all-InSb resonators, investigation of ultrahigh-Q factor TD resonances at terahertz frequencies arising from a distortion of symmetry-protected BIC in the metasurface^63^, nanoscale refractive index sensor with ultrahigh figure of merit based on toroidal dielectric metasurfaces which presents a novel approach to refractive index sensing using a dielectric metasurface with two semicircle disks clusters^1^, and …, where high sensitivity and selectivity for specific frequency bands were achieved.

In this paper, we proposed a highly-sensitive sensor with ultra-high QF and FOM based on the three method combinations, which includes (i) breaking the symmetry, (ii) design of complex structures, and (iii) changing the incidence wave angle, with the FR excitation in the designed structure. We have shown that with the arrangement of meta-atoms inside a meta-molecule of an all-dielectric material, we can simultaneously excite the electric and magnetic toroidal responses, as well as introduce a structure with a trapped (dark) mode that has the FR shape in the transmission spectrum. We enabled the quadrupole toroidal response by bound state in the continuum and for the first time, we were able to introduce and investigate a highly-sensitive sensor with an ultra-high QF in the quadrupole toroidal mode. Also, due to its very high S and QF, the proposed structure designed for measuring the RI changes in different environments (gas, bio and chemistry) can be very appropriate for sensing applications.

## Structure

**Figure 1:**
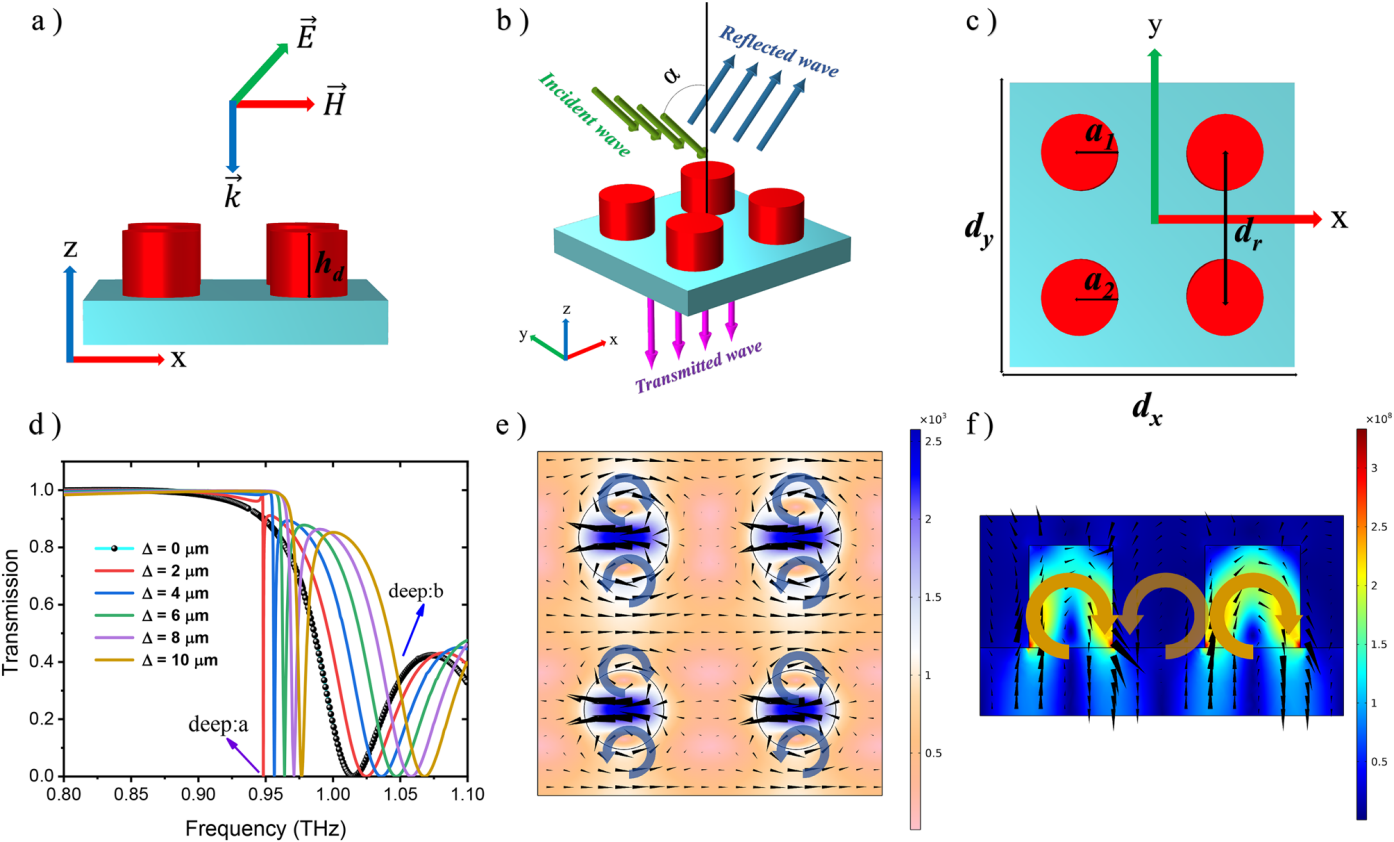
(**a**) The lateral view of the primary metasurface unit cell in the x–z plane consisting of four microdisks, with the input wave direction ***k*** = {0, 0, *k*_z_}and polarization ***E*** = {0, *E*_y_, 0}. (**b**) 3-D schematic of the metasurface unit cell; the green lines indicate the direction of the input wave with α angle to the normal axis, the blue lines indicate the direction of the reflected wave, and the purple lines indicate the direction of the transmitted wave from the metasurface structure. (**c**) The top cross-sectional view of the unit cell in the x–y plane; the periodicity in x and y directions is d_x_ = d_y_ = 320 µm, the radii of upper and lower disks are respectively a_1_ = 42 µm and a_2_ = a_1_–Δ, and the center-to-center distance between adjacent disks is d_r_ = 160 µm. (**d**) Transmission characteristics of the metasurface structure versus the frequency, for various values of Δ = a_1_–a_2_; the black line curve corresponds to *Tr* (*ω*) for the symmetric unit cell, i.e. Δ = 0. (**e**) The distribution of magnetic field for deep:b in the x–y plane. (**f**) The distribution of displacement current lines for deep:b in the y–z plane.

Figure 1 demonstrates the schematic of a primary metasurface unit cell consisting of four microdisks, where the radii of all disks are initially considered equal to each other. Figure 1a shows the lateral view of the unit cell, in which the height of the disks is *h*_*d*_ = 60 µm. In Fig. 1b, the 3-D schematic of the unit cell is observed, where the incident, reflection and transmission waves are illustrated. The top cross-sectional view of the unit cell is shown in Fig. 1c. As it is clear from this figure, the structure’s width and length in the x and y directions is *d*_*x*_ = *d*_*y*_ = 320 µm, while the radii of upper and lower disks are respectively *a*_1_ = 42 µm and *a*_2_ = *a*_1_ – Δ, where Δ is the difference between the radius of the upper and lower disks, and the center-to-center distance between adjacent disks is *d*_*r*_ = 160 µm. Also, the radius of each disk is determined according to the relation of *R*_*res*_ ≈ *c*/(2 *f*_*res*_* n*_*s*_)^64^. For the silicon resonators structure, the relative permittivity (dielectric constant) can be expressed from the Droud model as^65^

1 $$\varepsilon = {\varepsilon }_{\infty }-\frac{{\omega }_{p}^{2}}{{\omega }^{2}+j\omega \gamma },$$

where *ε*_*∞*_ = 11.7 is the permittivity of silicon at high frequencies, γ = 1.72 × 10^13^ rad/s is the collision frequency. Also, ω_*p*_ = (*ne*^2^⁄*m*_0_ ε_0_)^1/2^ is the plasma frequency, where *n* and *m*_0_ = 0.26 *m*_e_ are respectively doping concentration and effective mass of carriers. Additionally, SiO_2_ with the relative permittivity of *ε*_*r*_ = 3.75 and loss of tan*δ* = 4 × 10^–4^ is used as the substrate for silicon micro disks^66^. As Fig. 1 reveals, the metasurface structure is excited with the normal incident plane wave (*α* = 0º) at ***k*** = {0, 0, *k*_z_} and y-polarized electric field of ***E*** = {0, *E*_y_, 0}. For the symmetric structure i.e. *a*_1_ = *a*_2_, both *x* and *y* polarizations have identical transmission spectra, since symmetric structures have similar responses for a change in polarization in the propagation plane. The transmission spectrum of the proposed metasurface structure, *Tr* (ω) =|*S*_21_|, where *S*_*21*_ denotes the transmission term in scattering matrix, is calculated using the finite element method (FEM) as demonstrated in Fig. 1d. The black line curve represents *Tr* (ω) for the symmetric unit cell, i.e. Δ = 0, which has an ETD resonance deep at 1.012 THz. As this figure reveals, there are two deeps for different Δ values, one called deep:a and another deep:b, noting that there is only one deep for Δ = 0 (black line), which is deep:b. Figure 1e,f are corresponding to the distribution of magnetic field (in the x–y plane) and displacement current lines (in the y–z plane), respectively. The vortex displacement current in the y–z plane (Fig. 1f) with two tail to hail closed loop magnetic responses around each of the disks indicate an ETD response in each of them.

**Figure 2:**
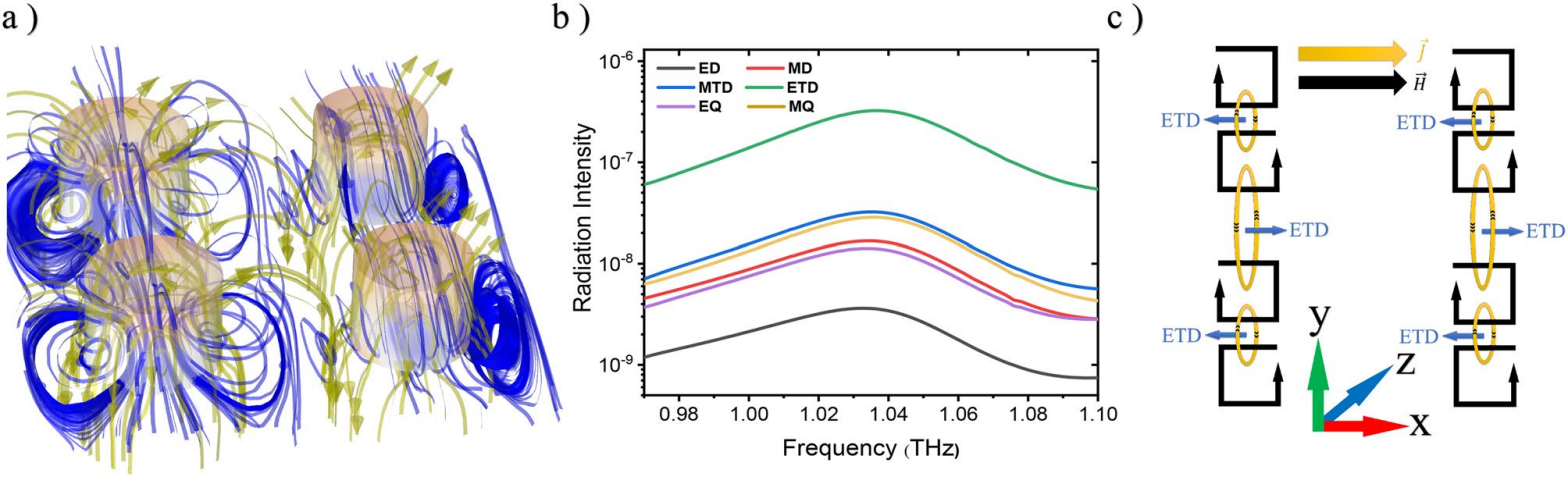
(**a**) 3D representation of magnetic field lines and displacement current at the ETD resonance mode for deep:b; blue and yellow ribbons correspond to magnetic fields and displacement currents, respectively. (**b**) Radiation intensities in terms of the frequency for deep:b. (**c**) 2D schematics for the formation of ETD moment and the rotation of the magnetic field; here, the yellow arrows show the poloidal current direction, the black loops demonstrate the magnetic vortex response and the blue arrows denote the direction of ETD moment.

Figure 2a illustrates the three-dimensional (3D) distribution of magnetic field lines (blue ribbons) and displacement currents (yellow ribbons) to better explain the ETD at the resonance frequency (deep:b). For calculating the mentioned scattered power of Mie multipoles, We can use^32,67–69^

2 $$ED: {P}_{\alpha }=\frac{1}{i\omega }\int {{\varvec{J}}}_{\boldsymbol{\alpha }} {d}^{3}r,$$

3 $$MD: {M}_{\alpha }=\frac{1}{2c}\int {({\varvec{r}}\boldsymbol{ }\times \boldsymbol{ }{\varvec{J}})}_{\boldsymbol{\alpha }} {d}^{3}r,$$

4 $$MTD: {T}_{\alpha }^{m}=\frac{1}{10c}\int \left[\left({\varvec{r}}\boldsymbol{ }.\boldsymbol{ }\boldsymbol{ }{\varvec{J}}\right) {r}_{\alpha }-2{r}^{2} {{\varvec{J}}}_{\alpha }\right] {d}^{3}r ,$$

5 $$ETD: {T}_{\alpha }^{e}=\frac{{\omega }^{2}}{20{c}^{2}}\int \left[{\left({\varvec{r}}\boldsymbol{ }\times \boldsymbol{ }\boldsymbol{ }{\varvec{J}}\right)}_{\boldsymbol{\alpha }} {r}^{2} \right] {d}^{3}r,$$

6 $$EQ: {Q}_{\alpha ,\beta }^{(e)}=\frac{1}{i2\omega }\int [{r}_{\alpha } {{\varvec{J}}}_{\beta }+\boldsymbol{ }{r}_{\beta } {{\varvec{J}}}_{\alpha }+ \frac{2}{3} {\delta }_{\alpha ,\beta } ({\varvec{r}} .\boldsymbol{ }\boldsymbol{ }{\varvec{J}})] {d}^{3}r,$$

7 $$MQ: {Q}_{\alpha ,\beta }^{(m)}=\frac{1}{3c}\int \left( {\left[{\varvec{r}} \times {\varvec{J}}\right]}_{\alpha }\boldsymbol{ }{r}_{\beta }+ {\left[{\varvec{r}} \times {\varvec{J}}\right]}_{\beta }\boldsymbol{ }{r}_{\alpha } \right) {d}^{3}r,$$

8 $$MTQ : {Q}_{\alpha ,\beta }^{(T)}=\frac{1}{28c}\int \left[4 {r}_{\alpha } {r}_{\beta } \left({\varvec{r}} . {\varvec{J}}\right)-5{r}^{2} \left({r}_{\alpha } {{\varvec{J}}}_{\beta }+ {r}_{\beta } {{\varvec{J}}}_{\alpha }\right)+2 {r}^{2} \left({\varvec{r}} . {\varvec{J}}\right) {\delta }_{\alpha ,\beta } \right]{d}^{3}r,$$

where *P*_*α*_, *M*_*α*_, ${T}_{\alpha }^{m}$, ${T}_{\alpha }^{e}$, ${Q}_{\alpha ,\beta }^{(e)}$, ${Q}_{\alpha ,\beta }^{(m)}$ and ${Q}_{\alpha ,\beta }^{(T)}$ are the scattering power of ED, MD, MTD, ETD, EQ, MQ and MTQ, respectively. Also, *c* is the speed of light in vacuum, ***r*** = (*x*, *y*, *z*) where *r*^2^ =|***r***^2^|= *x*^2^ + *y*^2^ + *z*^2^, *α* and *ꞵ* denotes one of the coordinates of *x*, *y* or *z*, and ***J*** represents the displacement current density, which can be calculated using^34^

9 $${\varvec{J}}={\varepsilon }_{0} \left(\varepsilon -1\right)\frac{d{\varvec{E}}}{dt}=i\omega {\varepsilon }_{0}\left(\varepsilon -1\right){\varvec{E}}$$

where *ω* is the angular frequency, *ε*_0_ is the free space permittivity, *ε* is the complex relative permittivity, and ***E*** is the electric field intensity. The radiation intensities in the far field corresponding to each of Eqs. (2)–(7) can be obtained by^70,71^

$${I}_{ED}=\frac{2{\omega }^{4}}{3{c}^{3}} {\left|{P}_{\alpha }\right|}^{2}$$, $${I}_{MD}=\frac{2{\omega }^{4}}{3{c}^{3}} {\left|{M}_{\alpha }\right|}^{2}$$, $${I}_{MTD}=\frac{2{\omega }^{4}}{3{c}^{3}} {\left|{T}_{\alpha }^{m}\right|}^{2}$$,$${I}_{ETD}=\frac{2{\omega }^{4}}{3{c}^{5}} {\left|{T}_{\alpha }^{e}\right|}^{2}$$.

10 $${I}_{EQ}=\frac{{\omega }^{6}}{5{c}^{5}} \sum {\left|{Q}_{\alpha ,\beta }^{(e)}\right|}^{2}, {I}_{MQ}=\frac{{\omega }^{6}}{20{c}^{5}} \sum {\left|{Q}_{\alpha ,\beta }^{(m)}\right|}^{2}, {I}_{MTQ}=\frac{{\omega }^{8}}{20{c}^{7}} \sum {\left|{Q}_{\alpha ,\beta }^{(T)}\right|}^{2} ,$$

where changes in terms of frequency can be observed in Fig. 2b. As this figure reveals, the ETD radiation intensity is stronger than the other multipoles. It is also worth mentioning that due to the symmetry of the metasurface unit cell, the MTQ is not excited inside the structure and consequently its intensity is very low, so it is not shown in this figure. To better understand the ETD resonance, the conceptual depiction of Fig. 2c has been drawn representing the magnetic field and displacement current lines to create the ETD mode. One way to excite a dark mode and achieve the FR is to break the symmetry in the metasurface by changing the radius of the lower disks through the change of Δ, which creates a new resonance mode at lower frequencies in addition to a shift towards higher frequencies in the transmission spectrum.

The full width at half maximum (FWHM) of deep:a was increased at high values of Δ, while it was decreased for deep:b, so Δ = 4 μm is selected to continue the simulation process. To reduce the FWHM in the metasurface structure, we can create defects inside the unit cell^35,72,73^. Noting that the FWHM for deep:a does not differ so much with the increase of defect number, while the minimum FWHM for deep:b occured when nine defects were applied in each disk, it was selected as the optimum structure. Table 1 illustrates FWHM values associated with deep:a and deep:b for various defect numbers. As is clear, the minimum FWHM value for deep:a and deep:b is obtained when four (quadruple) and nine defects emploed in each disk, respectively (Schematics and transmission curves for structures with 1 and 4 defects in Sect. 1 are shown).

**Table 1:** Comparison of FWHM values associated with deep:a and deep:b for various defect numbers.

Number of defects in each disk	FWHM for deep:a (THz)	FWHM for deep:b (THz)
1	0.0012	0.0165
3	0.00115	0.015
4	0.00112	0.0145
6	0.001135	0.0133
9	0.00115	0.0125
10	0.00117	0.0131

**Figure 3:**
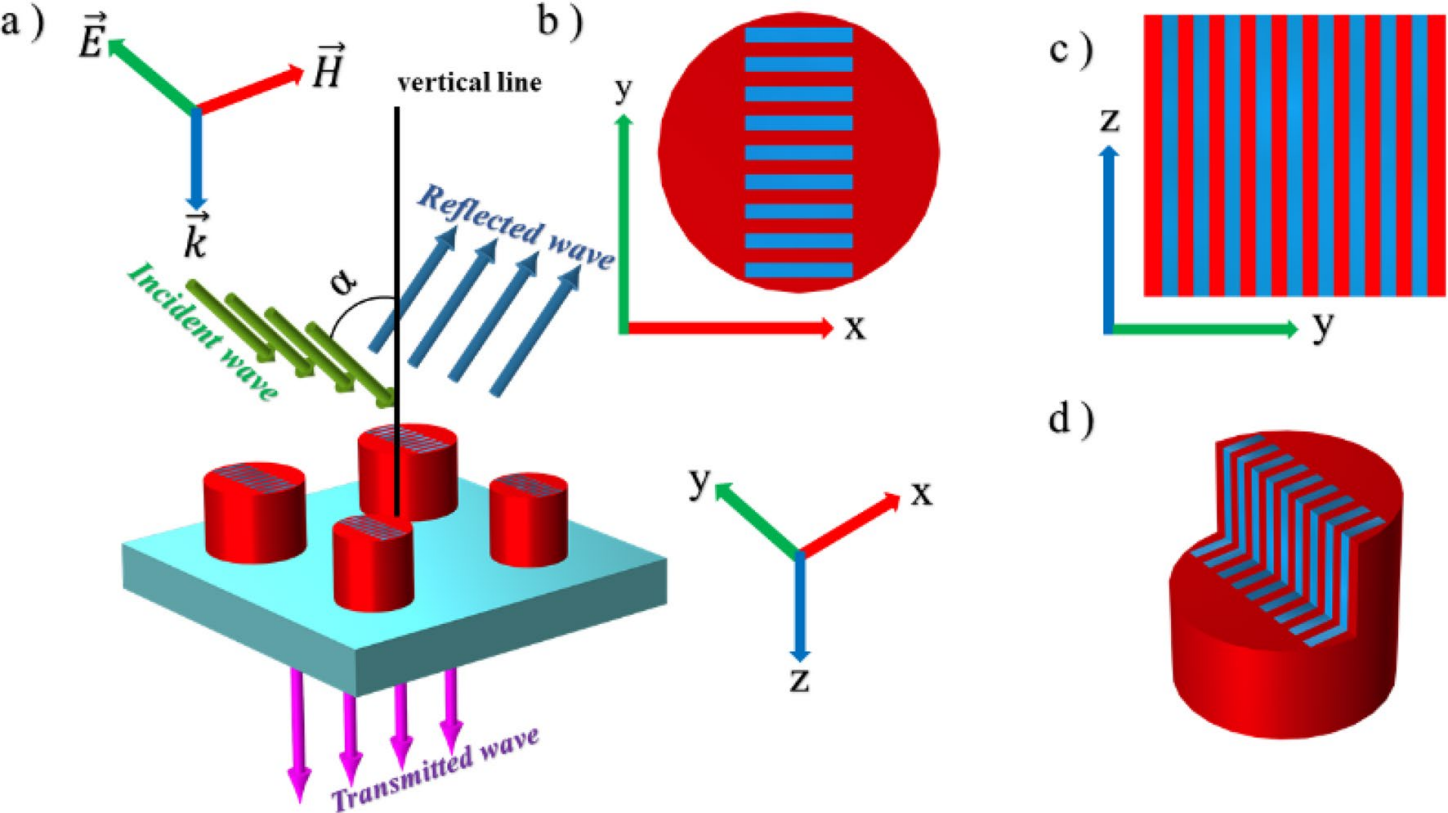
(**a**) 3D schematic view of the metasurface unit cell with nine defects in each of four silicon disks on a SiO_2_ substrate, where the incident wave (green lines) with an angle (α) to the vertical line, the reflected wave (blue lines) and the transmitted wave (purple lines) are specified. (**b**) Top cross-sectional view of grating like defect in the x–y plane. (**c**) Lateral cross-sectional view of grating like defect in the y–z plane. (**d**) 3D cross-sectional view of grating like defect in the y–z plane. The incident wave direction and polarization are considered as ***k*** = {0, 0, *k*_*z*_} and ***E*** = {0, *E*_y_, 0}, respectively.

**Figure 4:**
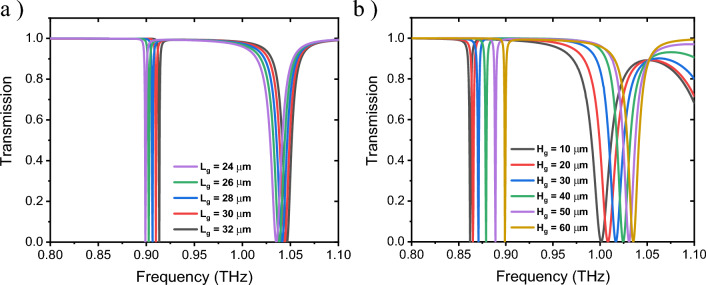
Transmission characteristics of the metasurface unit cell with the optimum width of defects equal to W_g_ = 4.4 μm, for (**a**) various lengths of defects, while H_g_ = 60 μm, and (**b**) different heights of defects, while L_g_ = 20 μm.

As explained above, the optimal design for the metasurface unit cell is when using nine defects in each of four silicon disks in the form of a grating in y-direction due to more interaction with the incident y-polarized electric field, i.e. ***E*** = {0, *E*_y_, 0}^52,74,75^. Figure 3a demonstrates the 3D schematic view of such optimum structure with the specified incident field with an angle (α) to the vertical line, as well as the reflected and transmitted fields. Figure 3b,c display the top and lateral cross-sectional views of grating like defect in the x–y and y–z planes, respectively. Also, for more clarification, a 3D cross-sectional view of grating like defect inside one silicon disk in the y–z plane can be observed in Fig. 3d. The width of each defect in upper disks is W_g_ = 4.4 μm and for lower disks is determined by W_gl_ = W_g_ × (a_2_/a_1_) to achieve the optimal structure. The related transmission spectra for different lengths and heights have been depicted in Fig. 4a,b, respectively. For the unit cell structure with single and quadruple defects, increasing L_g_ and H_g_ causes a frequency shift towards higher values, which will improve FWHM.

Considering that one of the other ways to excite the FR is changing the incidence angle, we changed this angle from 0 to 45 degrees, where a decrease in the FWHM of transmission spectrum and an increase in the interaction of the electric field with the resonator (silicon disks) were observed. Therefore, the sensor performance was improved.

**Figure 5:**
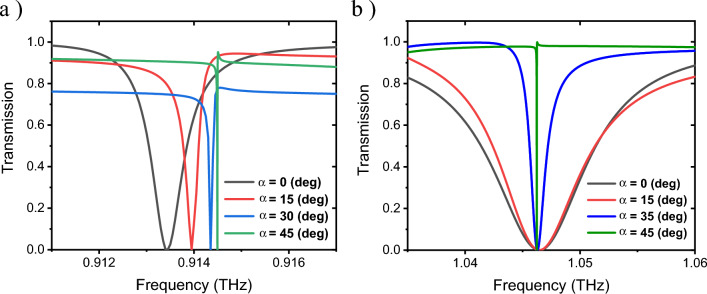
Transmission spectrum at different α incidence angle values from 0 to 45 degrees, corresponding to (**a**) deep:a and (**b**) deep:b.

**Figure 6:**
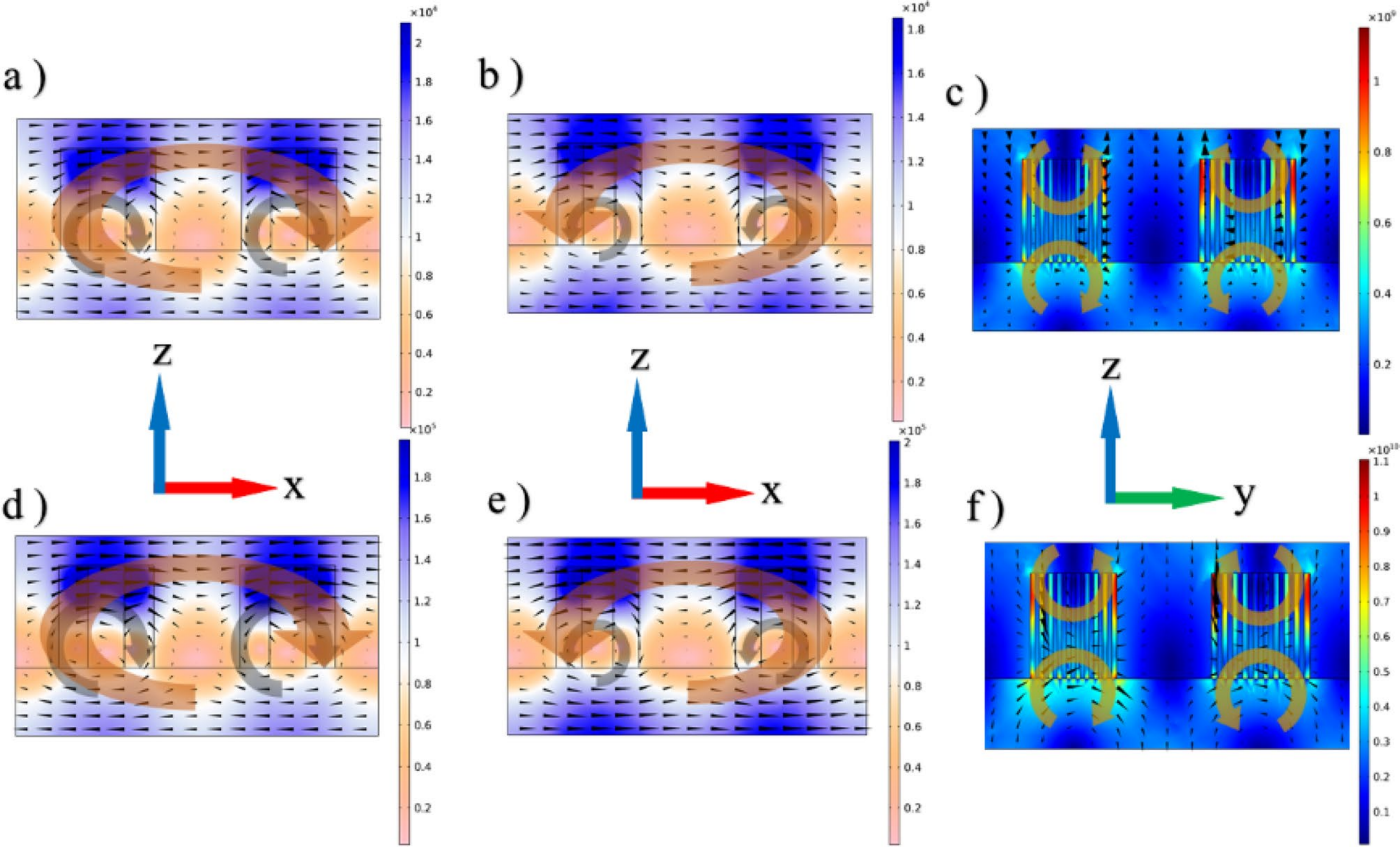
The distribution of the magnetic field created in the metasurface unit cell in the x–z (vertical) plane, for deep:a at α = 0°, for (**a**) the upper disks (with larger radius) and (**b**) lower disks (with smaller radius); where blacks cones indicate the propagation of magnetic field lines. (**c**) The distribution of the displacement current in the metasurface unit cell in the y–z plane, for deep:a at α = 0°. (**d**), (**e**) and (**f**) are similar to (**a**), (**b**) and (**c**), respectively, but at α = 45°.

Figures 5a,b display the transmission spectra in terms of the frequency for deep:a and deep:b with the incidence angle being changed from 0 to 45 degrees, respectively. It should be noted that since our main goal was to investigate the resonances in deep:a and deep:b, we have ignored other resonances that may occur due to the change of the incidence angle. Changing the incidence angle (α) increases the electromagnetic field confinement inside the silicon disks of the unit cell. Figure 6a,b shows the magnetic field distribution in the x–z (vertical) plane for upper disks at α = 0°, while Fig. 6d,e represents this distribution for lower disks at α = 45°. The displacement current distribution in the y–z plane is displayed in Figs. 6c,f at α = 0° and 45°, respectively.

Changing the incidence angle (α) increases the electromagnetic field confinement inside the silicon disks of the unit cell. Figure 6a,b show the magnetic field distribution in the x–z (vertical) plane for upper disks at α = 0°, whereas Fig. 6d,e represent this distribution for lower disks at α = 45°. The displacement current distribution in the y–z plane is displayed in Fig. 8c,f at α = 0° and 45°, respectively.

**Figure 7:**
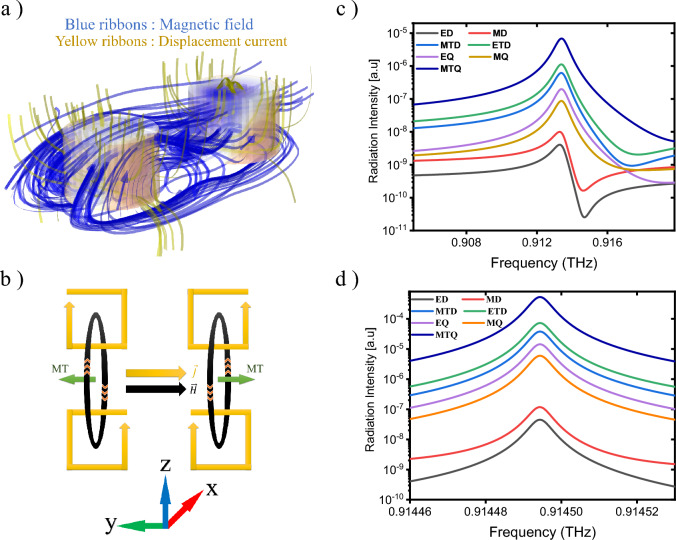
(**a**) 3D display for distribution of the magnetic field lines and displacement current to represent the MTD response at α = 45°. (**b**) 2D representation for the formation of MTQ response; where yellow loops indicate the direction of poloidal current, black loops show the magnetic vortex response and green arrows denote the direction of MTD moments. (**c**) and (**d**) demonstrate multipolar radiation intensities in terms of the frequency extracted from the Mie theory for deep:a at α = 0° and 45°, respectively.

Considering the behavior of the magnetic field and displacement current as depicted in Fig. 6a–f, a magnetic toroidal dipole (MTD) response is evident in each of the upper and lower disks, and a stronger MTD response is formed in the correlation between the upper disks according to Fig. 7a, which illustrates the 3D profiles of magnetic field (blue ribbons) and displacement current (yellow ribbons) in the upper disks at α = 45^°^. As this figure designates, a vortex magnetic behavior and closed-loop surface current have been formed in the magnetic field meridians. Also, as shown in Fig. 7b, the MTD response in the lower discs is in the opposite direction compared to the upper discs, the corresponding dipoles together form a strong MTQ. The radiation intensity characteristics due to Mie multipoles, which are obtained by solving Eqs. (2)–(10), as in Fig. 7c,d, also confirm a strong MTQ response at both α = 0° and 45°. (The electrical and magnetic response analysis and Fano resonance are shown in Sects. 2 and 3 respectively).

## Results and discussion

**Figure 8:**
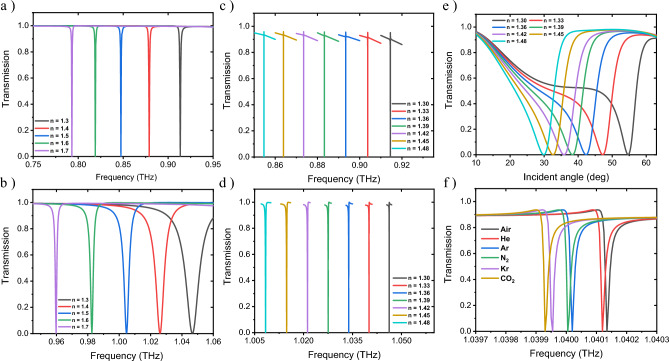
Transmission spectra in terms of the frequency (*Tr*(ω)) for different mediums (RIs) corresponding to (**a**) deep:a at α = 0°, (**b**) deep:a at α = 45°, (**c**) deep:b at α = 0° and (**d**) deep:b at α = 45°. Transmission spectra (**e**) in terms of the incidence angle α for various RI values, at the optimal frequency of 0.78 THz, and (**f**) in terms of the frequency for different gas environments, corresponding to deep:a at α = 45°.

Due to the importance of biomedical, chemical and gas electromagnetic sensors in recent years, sensor parameters for both deeps (deep:a deep:b) were calculated in various environments with different RIs. Also, the obtained results with previously reported works were compared. As Fig. 8a,b demonstrate, by changing the RI value from 1.3 to 1.7, a frequency shift is obtained in the transmission spectrum at α = 0° for deep:a and deep:b, respectively. Figure 8c,d display the corresponding spectra at α = 45° and for the RI range from 1.30 to 1.48.

According to Fig. 8, the best results were attained for S = Δ*f*/Δ*n*^76^, FOM = S/FWHM^77^ and QF = f_res_/FWHM^77^ as follows: (i) Deep:a at α = 0°; S = 345 GHz/RIU, FOM = 684 RIU^–1^ and QF = 1802. (ii) Deep:a at α = 45°; S = 357 GHz/RIU, FOM = 2.216 × 10^6^ RIU^–1^ and QF = 5.7 × 10^6^. (iii) Deep:b at α = 0°; S = 197 GHz/RIU, FOM = 217 RIU^–1^ and QF = 872. (iv) Deep:b at α = 45°; S = 210.4 GHz/RIU, FOM = 8244 RIU^–1^ and QF = 41,602. In order to evaluate the sensitivity of the designed structure to changes in the incidence angle α, at a constant frequency of 0.78 THz, which is the optimal frequency in terms of sensitivity to variations in α, the RI value was varied from 1.3 to 1.48. Additionally, the transmission spectrum was calculated for each RI as displayed in Fig. 8e. In this figure, the highest value for the angular sensitivity *S’* = Δα/Δ*n*^78,79^ was obtained, which is equal to 247 deg/RIU. Due to the high sensitivity and quality factor of the proposed sensor, it can be used in gas environmentsc^80^. As Fig. 8f demonstrates, frequency shifts per changes in the RI for different gases were occurred corresponding to deep: a at α = 45, in which the best result was achieved as S = 625 GHz/RIU, QF = 1.04 × 10^5^ and FOM = 6.25 × 10^4^ RIU^–1^.

Table 2 displays the changes of S, FOM and QF for deep:a and deep:b at α = 0° and α = 45°. Accordingly, the best result for the S value was achieved for deep:a at α = 45° and in a gas environment, and considering the strong response of MTQ compared to ETD at α = 45°, the best results for FOM and QF were also obtained for deep:a. (Sect. 4 shown S, FOM and QF changes versus the medium RI in unit cell metasurface, for deep:a and deep:b at α = 0° and α = 45°).

**Table 2:** Variations of S, FOM and QF; corresponding to deep:a and deep:b at different values of the incidence angle (α).

	Deep:a (α = 0°)	Deep:b (α = 0°)	Deep:a (α = 45°)	Deep:b (α = 45°)	Gas environments
S (GHz/RIU)	345	217	357	210.4	625
FOM (1/RIU)	684	197	2.21 × 10^6^	8244	6.25 × 10^4^
QF	1802	872	5.7 × 10^6^	41,602	1.04 × 10^5^

In Table 3, a comparison between the results of our work and previous reported works in the literature is represented. It can be concluded that according to the range of RI changes, the designed structure can be used well based on sensor parameters such as S, FOM and QF for sensing gas, bio and chemical environments. It should be recalled that for better comparison of the S values in Table 2, we used the following equation to convert the values in terms of GHz/RIU into the values in terms of nm/RIU^76^.

11 $$\left|\frac{d\lambda }{dn}\right|=\frac{c}{{f}_{0}^{2}}\times \frac{df}{dn},$$

where *c* is the speed of light in vacuum and *f*_0_ is the resonance frequency.

**Table 3:** Comparison between the results of our work and previous reported works.

Works	Resonator material	Frequency range	S	FOM	QF
2014^76^	Aluminum	THz	5.7 × 10^4^ nm/RIU	–	65
2019^77^	TiO_2_	Near IR	186.96 nm/RIU	721	5126
2021^1^	LiTaO_3_	THz	489 GHz/RIU	25,352	1.2 × 10^5^
2020^66^	Silicon	THz	77 GHz/RIU	11.1	–
2015^81^	Aluminum	THz	139.2 GHz/RIU	–	–
2021^82^	Gold	THz	–	20,000	–
2021^7^	Silicon	THz	16,042 nm/RIU	533	39,857
2022^83^	Gold	THz	458.3 GHz/RIU	–	15.2
This work deep:a (α = 45°)	Silicon	THz	625 GHz/RIU (1.73 × 10^5^ nm/RIU)	6.25 × 10^4^	1.04 × 10^5^
		Gas sensing			
This workdeep:a (α = 45°)	Silicon	THz	357 GHz/RIU (9.35 × 10^4^ nm/RIU)	2.21 × 10^6^	5.7 × 10^6^
		Chemical sensing			
This work deep:b (α = 45°)	Silicon	THz	210.4 GHz/RIU (6.27 × 10^4^ nm/RIU)	8244	41,602
		Biosensor			

## Method

FEM method with periodic boundary conditions along x and y axis is used for simulation. The incident field is along the z axis. The equations used in the FEM method are as follows:

12 $$\nabla \times \nabla \times {\overrightarrow{E}}_{s}-{k}_{0}^{2}{\varepsilon }_{r}{\overrightarrow{E}}_{s}=\overrightarrow{F,}$$

13 $$\overrightarrow{F}={k}_{0}^{2}\left({\varepsilon }_{r}-1\right){\overrightarrow{E}}_{inc},$$

14 $${\overrightarrow{E}}_{s}{|}_{{x}^{-}}={\overrightarrow{E}}_{s}{|}_{{x}^{+}}\times exp(-j{k}_{0}{d}_{x}{\text{sin}}{\theta }_{inc}{\text{cos}}{\varphi }_{inc}),$$

15 $${\overrightarrow{E}}_{s}{|}_{{y}^{-}}={\overrightarrow{E}}_{s}{|}_{{y}^{+}}\times exp(-j{k}_{0}{d}_{y}{\text{sin}}{\theta }_{inc}{\text{cos}}{\varphi }_{inc}),$$

where *E*_*s*_ and *E*_*inc*_ represent scattered and incident fields, respectively. Also, the dielectric relative permittivity and the free space wave number are represented by *ε*_*r*_ and *k*_*0*_, respectively. *d*_*x*_ is the spatial period along the x direction, while *d*_*y*_ is along the y direction. Moreover, *θ*_*inc*_ and *φ*_*inc*_ are defined as incident angles. Considering that FEM is a method based on field calculation, it is possible to calculate the amount of displacement current as mentioned in Eq. (9) by field calculation. One can use the differential form of Faraday's Law to calculate the magnetic field corresponding to a given electric field using Maxwell's equations. This involves solving the curl of the electric field to find the magnetic field. Ultimately, the resulted equation can be integrated to obtain the magnetic field.

### Feasibility of the structure

At first, the SiO_2_ substrates need to be cleansed. Then, a thin layer of intrinsic silicon can be fabricated on SiO_2_ using plasma-enhanced chemical vapor deposition (PECVD) method. After that, the wafer can be cleaned by acetone and deionized (DI) water for a specific amount of time. In the following, a suitable photoresist can be spin-coated on the wafer and then soft baked. The spin-coated layer can be then patterned into cylindrical arrays with rectangle arrays on the surface as illustrated in Sect. 4, followed by a photoresist development and a hard-baking process. Afterwards, a deep reactive ion etching (DRIE) method can be performed to etch the silicon. Finally, the remained photoresist can be cleaned using different liquids including acetone, isopropanol and DI water^84–86^.

## Conclusion

In summary, we designed an ultra-sensitive sensor with ultra figure of merit (FOM) and quality factor (QF) through the interaction of light with matter in ultra-sharp magnetic toroidal quadrupole and Fano resonance in the terahertz region. The sensor obtained using high QF resonance superficial in this work is better than other designs based on high-loss metamaterial and surface plasmonic resonance (SPR) structures. The designed sensor with strong MTQ response can be considered one of the best sensors in all-dielectric metasurface structures in the terahertz region in terms of S, QF, and FOM, compared to the reported values in recent years. We believe that the impact of our results in terahertz region could provide a way to design a super-sensitive chip in real-time.

### Supplementary Information

Supplementary Information.

## Data Availability

The data generated in this paper are available upon reasonable request from the corresponding author.
