# Supplementary material for: Refractive index sensor based on fano-magnetic toroidal quadrupole resonance enabled by bound state in the continuum in all-dielectric metasurface
Source: Sci Rep. 2024 Feb 19;14:4110. doi: 10.1038/s41598-024-54579-8 (PMC10876670; doi:10.1038/s41598-024-54579-8)
Supplement: Supplementary file 1 — Supplementary Information. [file 41598_2024_54579_MOESM1_ESM.docx]

**SUPPORTING INFORMATION FOR**

**Refractive Index Sensor based on Fano-Magnetic Toroidal Quadrupole Resonance Enabled by Bound State in the Continuum in All-dielectric Metasurface**

Javad Maleki^1^, Davood Fathi^1, *^

^1^ Department of Electrical and Computer Engineering, Tarbiat Modares University (TMU), Tehran, Iran

^*^ Corresponding author: d.fathi@modares.ac.ir

S1. Structure optimization

Figs. S1a, b & c demonstrate, we create a single defect in which the two upper disks have the length of L_g_, width of W_g_ and height of H_g_, whereas for the two lower disks, the length and width will be determined by L_gl_ = L_g_ × (a_2_ / a_1_) and W_gl_ = W_g_ × (a_2_ / a_1_), respectively. Fig. 2 displays the transmission spectrum of the metasurface unit cell for two configurations of Figs. 1a & d associated with single and quadruple defects created inside the resonators. Figs. S2a, b & c illustrates the transmission versus the frequency with a single defect as Fig. 1a for various changes in L_g_, W_g_ and H_g_, respectively. As is clear from these figures, with the increase of L_g_, W_g_ or H_g_, the resonance life-time increases and consequently the FWHM will be decreased in both deeps. In the next steps, we examine the enhancement of FWHM by increasing the number of defects in each of the unit cell discs, which in turn causes the electromagnetic field to be further confined and thus improves the sensing parameters of the designed structure. For increasing the number of defects, we create a quadruple defect in the form of a grating consisting of four defects with the same size and equal distance from each other, as shown in Figs. S1d & e. It should be mentioned that the sizes of the defects in the lower disks compared to the defects in the upper disks are reduced in proportion to the ratio of the radii of the lower and upper disks.

**Figure S1.** Schematic of defects created inside the metasurface unit cell: **(a)** 3D view of the unit cell structure with a single defect in the center of each disk; green, blue and purple arrows indicate respectively the incident, reflection and transmission waves. **(b)** Top view of a silicon disk with single defect of length L_g_ and width W_g_ in the x-y plane. **(c)** Cutting view of the disk with single defect in z-direction and height of H_g_. **(d)** 3D view of the unit cell structure with a quadruple defect. **(e)** Top view of a silicon disk with quadruple defect of length L_g_ and width W_g_ in the x-y plane.

Figs. S2d, e & f demonstrate the corresponding transmission spectra with quadruple defects which are respectively related to the change of length, width, and height of the defects, as in Fig. S1d. As these figures show, a shift toward higher frequencies occurs in *Tr* (ω) which is mainly due to the change of effective permittivity of the structure, and subsequently the FWHM will be improved owing to more confinement of the electromagnetic field inside the defects.

**Figure S2.** Transmission characteristics of the metasurface unit cell versus the frequency. With a single defect as Fig. 3a for various values of **(a)** L_g_ where W_g_ = 8 µm and H_g_ = 60 µm, **(b)** W_g_ where L_g_ = 20 µm and H_g_ = 60 µm, and **(c)** H_g_ where L_g_ = 20 µm and W_g_ = 8 µm; insets: zoom image of deep:b. With a quadruple defect as Fig. 3d for various values of **(d)** L_g_ where W_g_ = 4 µm and H_g_ = 60 µm, **(e)** W_g_ where L_g_ = 20 µm and H_g_ = 60 µm, and **(f)** H_g_ where L_g_ = 20 µm and W_g_ = 4 µm.

Section 2. Electrical and magnetic response analysis

For analyzing the electric and magnetic responses of metasurface structure at the resonance frequency using the effective relative permittivity and permeability, we use ^1–7^.

| $\varepsilon_{eff}= \frac{c}{j\pi fd}\times\frac{( 1- S_{21}-S_{11})}{( 1+ S_{21}+S_{11})}$ | (S.1) |
| --- | --- |
| $\mu_{eff}= \frac{c}{j\pi fd}\times\frac{( 1- S_{21}+S_{11})}{( 1+ S_{21}-S_{11})}$ | (S.2) |

where S_11_ and S_21_ are respectively the reflection and transmission components of the S matrix, where S_11_ = 1 - S_21_ due to very low absorption of silicon and SiO_2_ in the terahertz region, and *d* is the resonator thickness. As Fig. S3a (pink line) which is associated with *μ_eff_* variations shows, we have a strong magnetic response in deep:a with the deep value of *μ_eff_* = -60 in the resonance frequency. Also as the orange line reveals in this figure, since the *ε_eff_* curve has no changes in the resonance frequency, we have no electric response in deep:a. The optimal structure is with L_g_ = 32 μm, W_g_ = 4.4 μm and H_g_ = 60 μm, which the corresponding transmission characteristics (amplitude – blue line & phase – green line) associated with deep:a and deep:b have been illustrated in Figs. S3a & b, respectively. As is clear, a 180-degree phase break is observed in the transmission spectra of two mentioned deeps. Regarding deep:b as Fig. S3b illustrates, we have both electrical (orange line) and magnetic (pink line) responses in the metasurface unit cell. However, since the responses in this deep is weaker than those of deep:a, as Figs. S3a represent, the FWHM value for deep:b is greater than that of deep:a.

**Figure S3**. Transmission characteristics (amplitude – blue line & phase – green line), effective permittivity (orange line) and effective permeability (purple line), for the optimal structure with Lg = 32 μm, W_g_ = 4.4 μm and H_g_ = 60 μm, associated with **(a)** deep:a and **(b)** deep:b.

Section 3. Fano resonance analysis

To calculate the Transmission in the metasurface structure that has a spectral shape of the FR, we can use the coupled oscillator model ^8,9^

| $T_{Fano}=C {\frac{(\zeta+q )}{\zeta^{2}+1}}^{2}$ | (S.3) |
| --- | --- |

which *C* is the normalized coefficient, *q* is a constant parameter, and $\zeta=(\omega-\omega_{F})/\gamma_{F}$ where 𝜔*_F_* is the resonance frequency and *γ_F_* is the damping factor. Fig. S4 shows that the above equation can well reproduce the result obtained from the theory (FEM), which means that the resonance at the metasurface is justified by the coupled oscillator model.

**Figure S4:** Comparing the transmission spectrum associated with deep:a at α = 45°, calculated by (i) the theory (FEM) and (ii) the FR using equation (S.3).

Section 4: Results

Fig. S5 displays the S, FOM and QF changes versus the medium RI in unit cell metasurface, for deep:a and deep:b at α = 0° and α = 45° , in all the relevant sub-figures the RI of 1.3 has been considered as a comparison reference. As is clear from the sub-figures of the first column, the best performance for the S parameter is attained for deep:a at low RI values, while it is obtained for deep:b at higher RIs, It is obvious from the sub-figures of the second column that the best performance for the FOM parameter is achieved for deep:a at high RI values for both α = 0° and 45° (due to the decrease of FWHM), whereas for deep:b the best performance is reached at high RIs for α = 0° (because of the reduced FWHM) and lower RIs for α = 45° (owing to the increase of FWHM). For the third column sub-figures we find the same behavior as the sub-figures in the second column, but for the QF parameter.

**Figure S5.** Variations of S, FOM and QF versus the medium RI, respectively corresponding to **(a)**, **(b)** and **(c)** deep:a at α = 0^°^; **(d)**, **(e)** and **(f)** deep:b at α = 0^°^; **(g)**, **(h)** and **(i)** deep:a in α = 45^°^; **(j)**, **(k)** and **(l)** deep:b at α = 45^°^.

Section 5. Feasibility of the structure

Overview of Feasibility of the structure is shown in Fig. S6.

**Figure S6**. Overview of Feasibility of the structure.

References:

1. Zhao, Q., Zhou, J., Zhang, F. & Lippens, D. Mie resonance-based dielectric metamaterials. *Mater. Today* **12**, 60–69 (2009).

2. Hasan, M. M., Faruque, M. R. I. & Islam, M. T. Compact left-handed meta-atom for S-, C- and Ku-band application. *Appl. Sci.* **7**, (2017).

3. Dehbashi, R., Fathi, D., Mohajerzadeh, S. & Forouzandeh, B. Equivalent left-handed/right-handed metamaterial’s circuit model for the massless Dirac fermions with negative refraction. *IEEE J. Sel. Top. Quantum Electron.* **16**, 394–400 (2009).

4. Hajizadegan, M., Fathi, D. & Sakhdari, M. S. All-optical metamaterial switch based on Kerr effect with MWCNT composite. *Phys. E Low-dimensional Syst. Nanostructures* **48**, 1–6 (2013).

5. Nguyen, H. A. *et al.* Tunable dynamic metamaterial for negative refraction. *J. Phys. Chem. Solids* **186**, 111804 (2024).

6. Kumar, D. *et al.* Photoinduced dynamic tailoring of near-field coupled terahertz metasurfaces and its effect on Coulomb parameters. *J. Opt.* **24**, 45101 (2022).

7. Sim, J. & Zhao, R. R. Magneto-Mechanical Metamaterials: A Perspective. *J. Appl. Mech.* **91**, (2024).

8. Srivastava, Y. K. *et al.* Fano resonances in terahertz metasurfaces: Strong influence of metallic conductivity at extremely low asymmetry. Adv. *Opt. Mater* **4**, 457–463 (2016).

9. Chen, S., Jin, S. & Gordon, R. Subdiffraction focusing enabled by a Fano resonance. *Phys. Rev. X* **4**, 1–8 (2014).
